# Refining tacrolimus dosing through CYP3A5 pharmacogenetics in Taiwanese renal transplant recipients

**DOI:** 10.1080/0886022X.2025.2523567

**Published:** 2025-09-11

**Authors:** Yen-Lin Chang, Tzu-Hung Hsiao, Yi-Ming Chen, Ching-Heng Lin, Yi-Ju Liao, Chiann-Yi Hsu, Yung‑Po Liaw, Cheng-Hsu Chen

**Affiliations:** ^a^ Department of Pharmacy, Taichung Veterans General Hospital, Taichung, Taiwan; ^b^ Department of Public Health and Institute of Public Health, Chung Shan Medical University, Taichung City, Taiwan; ^c^ Department of Medical Research, Taichung Veterans General Hospital, Taichung, Taiwan; ^d^ Department of Public Health, College of Medicine, Fu Jen Catholic University, New Taipei City, Taiwan; ^e^ Institute of Genomics and Bioinformatics, National Chung Hsing University, Taichung, Taiwan; ^f^ Division of Allergy, Immunology and Rheumatology, Department of Internal Medicine, Taichung Veterans General Hospital, Taichung, Taiwan; ^g^ Institute of Biomedical Science and Rong Hsing Research Center for Translational Medicine, National Chung Hsing University, Taichung, Taiwan; ^h^ School of Medicine, National Yang Ming Chiao Tung University, Taipei, Taiwan; ^i^ Department of Post-Baccalaureate Medicine, College of Medicine, National Chung Hsing University, Taichung, Taiwan; ^j^ Department of Health Care Management, National Taipei University of Nursing and Health Sciences, Taipei, Taiwan; ^k^ Department of Industrial Engineering and Enterprise Information, Tunghai University, Taichung, Taiwan; ^l^ Institute of Public Health and Community Medicine Research Center, National Yang Ming Chiao Tung University, Taipei, Taiwan; ^m^ Department of Medical Research, China Medical University Hospital, Taichung, Taiwan; ^n^ Department of Pharmacy, National Yang Ming Chiao Tung University, Taiwan; ^o^ Biostatistics Task Force of Taichung Veterans General Hospital, Taichung, Taiwan; ^p^ Institute of Medicine, Chug Shan Medical University, Taichung City, Taiwan; ^q^ Department of Medical Imaging, Chung Shan Medical University Hospital, Taichung City, Taiwan; ^r^ Division of Nephrology, Department of Internal Medicine, Taichung Veterans General Hospital, Taichung, Taiwan; ^s^ Department of Life Science, Tunghai University, Taichung, Taiwan

**Keywords:** Tacrolimus, CYP3A5, pharmacogenetics, taiwanese population, Renal transplantation

## Abstract

Genetic polymorphisms in CYP3A5 affects tacrolimus (Tac) bioavailability; however the optimal genotype-guided starting dose remains undefined. This retrospective cohort study examined the effects of the CYP3A5 allele on Tac pharmacokinetics in 431 Taiwanese renal transplant recipients. Genotyping was performed using the Axiom Genome-Wide TWB 2.0 array, with dose requirements and blood concentrations analyzed at multiple postoperative time points. CYP3A5 *3/*3 carriers required the largest dose reductions and exhibited the highest rate of Tac overexposure events (34.14%). Based on these results, we back-calculated an initial dosing formula to demonstrate that reducing doses for poor metabolizers (PMs) using a genotype-guided approach in our population could lower the risk of overexposure. Although preliminary, a genotype-guided initial dosing strategy reduced the likelihood of Tac overexposure by 69% (OR = 0.307, *p = 0.018*). These findings highlight the importance of identifying CYP3A5 poor metabolizers (*3/*3) for personalized therapy to minimize the risk of overexposure. Preemptive pharmacogenetic testing shows promise for enhancing dosing precision and treatment safety in Taiwanese patients.

## Introduction

Tacrolimus (Tac) is a global cornerstone of immunosuppressive therapy after kidney transplantation (KTx) [1,2]. However, its clinical use presents challenges owing to its toxicity, narrow therapeutic window, and high interindividual pharmacokinetic variability [3]. Prompt achievement of effective therapeutic blood levels after transplantation is critical for preventing organ rejection [4]. The initial dose after KTx is a key determinant [5–9], yet it often takes 2–3 weeks to adjust the starting dose to a stable maintenance level *via* therapeutic drug monitoring (TDM) [9]. Consequently, many patients experience overdosing during the early post-transplant phase [10–12]. Older patients, in particular, face a 43% risk of overexposure, often associated with a high rate of delayed graft function [13].

CYP3A5, an enzyme central to Tac metabolism, exhibits single nucleotide polymorphisms (SNPs) that account for 40–50% of variability in Tac blood concentrations [1,14,15]. Allele frequencies of CYP3A5 vary across ancestries [16–18]. In Caucasians and Asians, nonfunctional CYP3A5 alleles (*3, *6, *7) predominate, primarily as CYP3A5*3, while the CYP3A5*1/*1 genotype (conferring extensive metabolism) occurs in approximately 1% of Caucasians and 5–15% of Asians [17,19]. In contrast, the CYP3A5*1 allele is more prevalent in individuals of African descent, with frequencies ranging from 45–73%, contributing to a higher proportion of extensive metabolizers [17]. As a result, Asians and Africans are more likely than Caucasians to require higher Tac doses to avoid underexposure.

Various professional societies, such as the Clinical Pharmacogenetics Implementation Consortium (CPIC) [19], the Royal Dutch Association for the Advancement of Pharmacy-Pharmacogenetics Working Group (DPWG), and French National Network of Pharmacogenetics (RNPGx) [20], recommend individualizing Tac treatment based on known CYP3A5 genotypes. CYP3A5 phenotypes were clustered according to the CYP3A5*3 allelic status: poor (PMs) (CYP3A5*3/*3), intermediate (IMs) (CYP3A5*1/*3) and extensive metabolizers (EMs) (CYP3A5*1/*1 carriers).

The CPIC guideline recommends increasing the starting dose by 1.5–2.0 times the standard amount for IMs and EMs, consistent with RNPGx annotations, while the DPWG suggests a 1.5- and 2.5-fold increase for IMs and EMs, respectively. However, most supporting evidence is derived from Caucasian populations and Western societies, leaving genotype-guided Tac dosing for Han Chinese populations underexplored.

We conducted a retrospective study to investigate the association between CYP3A5 alleles and Tac concentrations in the Taiwanese population. We found that, in this study population, the optimal strategy was not to increase the initial dose for IMs and EMs but to reduce the initial dose for PMs, in contrast to the guideline recommendations. Based on our results, we back-calculated a preliminary Tac dosing formula for Taiwanese patients, which reduced Tac overexposure by 69% in this cohort, offering the potential to enhance treatment precision and safety.

## Materials and methods

### Subjects

From June 2019 to November 2022, we recruited patients over 20 years of age from a medical center in Taiwan, in cooperation with the Taiwan Precision Medicine Initiative (TPMI) project, which is managed by Academia Sinica, Taiwan. Clinical information on the participants was collected using medical records, and all participants were genotyped with an Affymetrix TWB 2.0 SNP array. In this retrospective study we included patients who: (1) underwent KTx for the first time; (2) were on a Tac-based immunosuppressive regimen and (3) had CYP3A5 gene information (6986 G > A, rs776746) from TPMI and were retained after quality control.

Patients received a Tac-based immunosuppressive regimen, typically combined with mycophenolate mofetil (MMF) or azathioprine and corticosteroids (prednisone). The standard protocol included an initial Tac dose of 0.15–0.2 mg/kg/day, MMF at 1–2 g/day or azathioprine at 1–2 mg/kg/day, and prednisone starting at 20 mg/day, tapered to 5–10 mg/day by 3 months post-transplant. Patients were administered either immediate-release Tac (Prograf^®^) or extended-release Tac (Advagraf^®^), based on clinical judgment and patient-specific factors. The target concentration of the pre-dose Tac trough was 8 to 12 ng/mL three months after KTx. The dose-adjusted Tac trough concentration (C/D) was calculated by dividing the measured C by the corresponding daily weight -adjusted Tac dose (ng/ml per mg/kg).

### Definition of outcomes

The primary outcome was the occurrence of Tac overexposure (12 < *C* < 20 ng/mL) and Tac at toxic levels (C> =20 ng/mL) as seen in our cohort within a 3-month period. Intra-individual variability (IIV) in Tac doses or concentrations was expressed as a coefficient of variation using the following formula: CV% = (σ/μ) × 100. IIV was calculated from at least three Tac levels measured within the first 90 days after transplant, using routine TDM values closest to days 0, 10, 30, 60, and 90, depending on clinical availability. These analyses were performed separately for each group of genotypes of CYP3A5.

### Building a dosing equation

To personalize Tac dosing, we conductedunivariate linear regression analysis to assess the association between the 3-month dose-adjusted Tac trough concentration (C/D ratio) and CYP3A5 genotypes in 253 renal transplant recipients with available body weight data. Patients were randomly divided into training (*n* = 168) and validation (*n* = 85) cohorts. The training cohort’s regression results are shown in Supplementary Table 1. A dosing equation (Equation 1) was derived to estimate the required Tac dose (mg/kg/day) to achieve target trough concentrations (8–12 ng/mL) during the initial 3-month post-transplant period.

### Statistical analysis

Categorical variables were summarized as frequencies and percentages. Continuous variables are summarized as mean and standard deviation (SDs) or median and IQR. The Kruskal-Wallis test was used to compare continuous Tac measurement variables among the three groups. All statistical analyses were performed using SPSS^®^ statistical software package (version 22.0, IBM Inc., Armonk, NY, United States). Statistical significance was set at *p* < 0.05 was considered statistically significant.

## Results

### Genotyping and patient characteristics

In total, 431 KTx recipients were enrolled in this study. Clinical characteristics and genotype frequencies are summarized in Table 1. The patients had a mean age of 42.1 years old and were predominantly male (231/431; 53.6%). The mean body mass index was 23.6 kg/m^2^. The number of genotype CYP3A5 *3/*3, *1/*3, and *1/*1 were 224 (52.0%), 162 (37.6%), 45 (10.4%), respectively.

**Table 1.** Baseline patient characteristics and genotype frequencies.

Characteristics	All treated patients(*n* = 431)	
Sex, n(%)		
Female	200	(46.4%)
Male	231	(53.6%)
Age (years), mean ± SD	42.1 ± 13.2	
BMI (kg/m^2^), mean ± SD	23.6 ± 3.6	
Creatinine (µmol/L), mean ± SD	2.19 ± 1.88	
Baseline immunosuppression, n(%)		
Prednisone, n(%)	257	(59.6%)
Azathioprine, n(%)	3	(0.7%)
MMF, n(%)	169	(39.2%)
CYP3A5 rs776746, n(%)		
*1/*1	45	(10.4%)
*1/*3	162	(37.6%)
*3/*3	224	(52.0%)

The allele frequencies of CYP3A5 *1 in our cohort (29.23%) were similar to those in the TPMI (28.80%) and gnomAD database (28.16%) [21].

### CYP3A5 genotype were associated tac dose, C/D ratio and overexposure ratio

**Figure 1.**
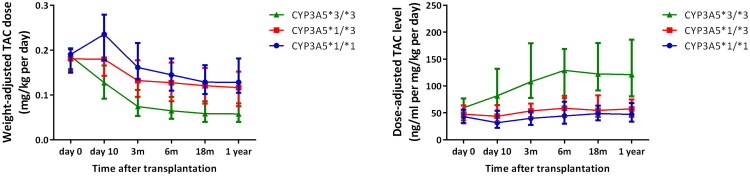
Distributions of weight-adjusted tac dose(A) and the C/D (B) of the individuals with *1/*1 or *1/*3 or *3/*3 CYP3A5 genotypes at day 0, day 10, 3 month, 6 months, 9 months, and 1 year.

There was a significant correlation between the CYP3A5 genotype and Tac dose. The recipients were classified into three groups according to their CYP3A5 genotype (*1/*1, *1/*3, and *3/*3) to explore the association between Tac pharmacokinetics and different alleles. The initial dose for all recipients did not exceed 0.1–0.2 mg/kg/day, which is recommended by the drug labels approved by the US Food and Drug Administration (FDA). As shown in Figure 1(A), the weight-adjusted Tac dose was significantly lower in CYP3A5 *3/*3 than in CYP3A5 *1/*1 carriers and CYP3A5 *1/*3carriersat day 10, 3momths, 6 months, 9 months and 1 year after transplantation (*p* < 0.01). The Tac C/D ratio also showed significant differences among the three groups at all -time points. The C/D ratio of CYP3A5 *3/*3 carrierswas the highest among the three groups, with the C/D ratio of CYP3A5 *1/*1 carrier being the lowest (Figures 1(B)). The detailed data are shown in Supplementary Table 2. Our data also showed that CYP3A5 *3/*3 carriers experienced a higher rate of overexposure (34.14%, *p* < 0.01) (Table 2). However, CYP3A5 polymorphism did not affect Tac intra-individual variability (IIV) at the dosage or concentration level.

**Table 2.** Patient pharmacokinetic values during the study period according to CYP3A5 genotype.

	CYP3A5 in B1-22						*p* value
	CYP3A5*1*1 (*n* = 45)		CYP3A5*1*3 (*n* = 162)		CYP3A5*3*3 (*n* = 224)		
Within 3 months							
Tac concentration overexposure (12 < *C* < 20 ng/mL)	5	(11.11%)	25	(15.43%)	72	(32.14%)	<0.001**
Tac toxic concentration (> =20 ng/mL)	2	(4.44%)	1	(0.62%)	10	(4.46%)	0.078
Intra-individual variability (IIV) of Tac							
Within 3 months (0–90 day)	18.33	(3.13–25)	25.00	(12.5–25)	20.00	(14.29–25)	0.192
Intra-individual variability (IIV) of Tac concentration							
Within 3 months (0–90 day)	25.00	(20–33.33)	33.33	(20–33.33)	25.00	(20–33.33)	0.481

Chi-Square test. Kruskal Wallis test. *p < 0.05, **p < 0.01. Result are expressed in median(Q1-Q3) or as number and (percentage).

### Initial dose adjusted downward after three months

**Figure 2.**
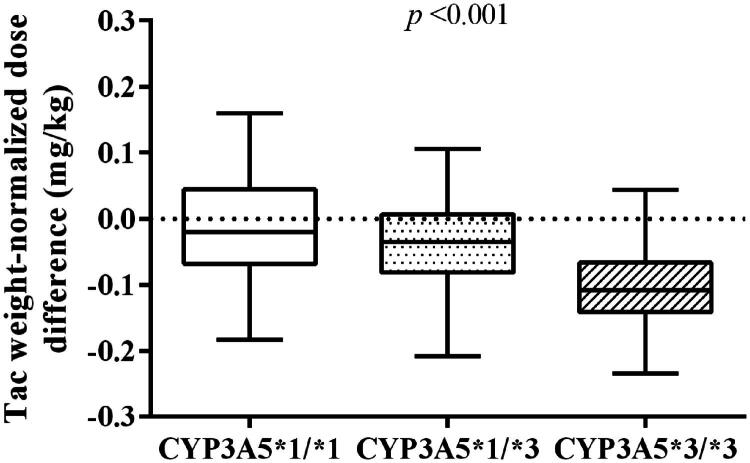
Comparison of body weight-adjusted dosage of tacrolimus within 3 months after surgery between CYP3A5 genotype. The box spans data between two quartiles (IQR), with the median represented as a bold horizontal line.

In order to understand the changes in the starting dose in different CYP3A5 genotypes, we compared the Tac dose from day0 and 3 months. We found that all participants, regardless of phenotype, had decreased doses after 3-month (*p* < 0.001), respectively (Figure 2). The Tac median dose was adjusted down to 0.16, 0.13, and 0.07 mg/kg/day in different CYP3A5 genotype after three months. Our data also showed that the CYP3A5 *3/*3 carriershave the highest degree of decline, which decrease from 0.19 to 0.07 mg/kg/day (63%).

### Optimize tac dosing strategy through regression model

We performed linear regression analysis to determine the association of 3 months Tac C/D ratio. A Tac genotype-guided initial dosing strategy to calculate the required Tac dose/kg to attain the desired target Tac level during the initial post-transplant period was developed as Equation 1 (Eq.1).

Equation 1 Required Tac dose/kg/daily =

(1) Desired stable tacrolimus level(ng/mL)111−70(CYP3A5∗1∗1)−55(CYP3A5∗1∗3)

CYP3A5*1/*1 or *1/*3 genotypes were coded as 1 (expressors), and CYP3A5*3/*3 as 0 (non-expressors).

The univariate model explained 27.6% of Tac dose variability (R^2^ = 0.276, *p* < 0.001; Supplementary Table 1). To explore potential confounders, a multivariate model including sex as a covariate was tested but showed no significant contribution (*p* = 0.153), with R^2^ increasing marginally from 0.276 to 0.281. The univariate model was retained for its simplicity and clinical applicability. In the validation cohort (*n* = 85), Eq.1 reduced Tac overexposure risk (>12 ng/mL) by 69% (OR = 0.307, *p* = 0.018). An expected initial Tac dosing table based on Eq.1 is provided in Table 3.

**Table 3.** Ranges of expected initial tac daily dose (mg/kg) based on CYP3A5 genotype.

	Target concentration (ng/ml)				
CYP3A5 genotype	8	9	10	11	12
CYP3A5*1/*1	0.20	0.22	0.24	0.27	0.29
CYP3A5*1/*3	0.14	0.16	0.18	0.20	0.21
CYP3A5*3/*3	0.07	0.08	0.09	0.10	0.11

## Discussion

In our study, there was a significant correlation between the CYP3A5 genotype and Tac dose. After 3 months, CYP3A5 *3/*3 carriers experienced the greatest reduction in prescribed Tac dose and the highest percentage of Tac overexposure events (34.14%). Consequently, we suggest that the starting dose for KTx recipients should be lowered for carriers of CYP3A5 *3/*3. We propose starting doses of 0.20–0.29 mg/kg/day for CYP3A5 1/1 carriers, consistent with current practice, 0.14–0.21 mg/kg/day for CYP3A5 *1/*3, and 0.07–0.11 mg/kg/day for CYP3A5 *3/*3, adjusted downward to maintain the therapeutic range of 8–12 ng/mL. Although preliminary, our genotype-informed dosing strategy, back-calculated from this Taiwanese cohort, reduced Tac overexposure risk by 69% within 3 months (OR = 0.307, *p = 0.018*). These findings indicate that preemptive pharmacogenetic testing offers potential for optimizing Tac therapy and enhancing treatment safety in Taiwanese renal transplant recipients, subject to further refinement and prospective validation.

Populations with a high prevalence of the CYP3A5*1 allele, such as African Americans, typically exhibit rapid Tac metabolism due to increased CYP3A5 enzyme activity. The Clinical Pharmacogenetics Implementation Consortium (CPIC) recommends increasing the initial Tac dose by 1.5–2 times for CYP3A5*1/*1 or *1/*3 carriers to achieve therapeutic trough concentrations (8–12 ng/mL) [19]. Conversely, our Taiwanese cohort, predominantly CYP3A5*3/*3 (poor metabolizers), exhibited slower Tac metabolism, with 34.14% experiencing overexposure (>12 ng/mL). Lower doses (0.07–0.11 mg/kg/day) were necessary to prevent toxicity. These findings indicate the necessity of population-specific Tac dosing strategies.

Although CYP3A5 is the primary determinant of Tac metabolism, CYP3A4 polymorphisms, notably CYP3A4*22 (rs35599367, C > T), also influence pharmacokinetics [22]. The CYP3A4*22 allele reduces enzyme activity by decreasing mRNA expression, resulting in higher dose-adjusted Tac trough concentrations (C/D ratio) and reduced dose requirements, particularly in CYP3A5*3/*3 carriers. In our Taiwanese cohort, where CYP3A5*3/*3 patients had the highest C/D ratios and required the lowest doses (0.07–0.11 mg/kg/day), CYP3A4*22 could theoretically increase overexposure risk. However, its prevalence in Asian populations is low (<1%) [23], and CYP3A5 dominates Tac metabolism. Thus, our study prioritized CYP3A5 genotyping (rs776746, 6986 G > A). Future studies integrating CYP3A4*22 and CYP3A5 polymorphisms could refine genotype-guided dosing, especially in populations with higher CYP3A4*22 frequencies, such as Caucasians.

Numerous studies have explored genotype-phenotype associations to improve KTx outcomes [24,25]. Significant associations have been reported between CYP3A5 and the Tac doses required for KTx recipients [26]. Our data also show that the Tac dose requirement was 1.5-fold to 2-flod higher in CYP 3A5 *1/*1 carriers than in CYP 3A5*1/*3 and CYP 3A5*3/*3 carriers. Dose-normalized trough levels (ng/mL/mg total daily dose, C/D ratio) as a predictive marker could help estimate an individual’s Tac metabolism [27–29]. Our data showed that the mean C/D value was higher than that in other Asian studies [6,30], either in CYP3A5 expressers (CYP3A5 *1 carriers) or non-expressers (CYP3A5 *3/*3). This suggests that our patient’s overall metabolic rate of CYP3A5 may have been slower than that of other patients. Therefore, if recipients treated according to current practices did indeed have high rates of overexposure or toxic concentrations, they could be associated with nephrotoxicity, neurotoxicity and new-onset diabetes after transplantation, as well as the onset of malignancies or infections. To avoid potentially harmful Tac overexposure, precision medicine targets the adjustment of prescriptions based on information about genetic polymorphisms.

In a randomized controlled study that included 280 KTx recipients, Thervet*et al [*15]. showed that adaptation based on CYP3A5 (with 2 dose regimens, 0.3 mg/kg/day for CYP3A5 expressers and 0.15 mg/kg/day for CYP3A5 non-expressers) of the Tac initial dose was associated with not only a higher proportion of patients achieving target C_0_ at an earlier time post-transplantation, but also fewer Tac dose modifications and a shorter delay in reaching target C_0_ levels when compared to simple weight-based dosing in which all patients received a fixed dose of 0.2 mg/kg/day. Therefore, dosing schedules based on the conclusions of Thervetet al. were further translated into the recommendations of the CPIC [19].

However, in 2016, another randomized controlled trial involving 237 KTx recipients showed that there was no significant difference between a genotype-based dose group and a standard dose group (*p* = 0.76) in Tac exposure within the therapeutic range (10–15 ng/ml) at their first steady state (day 3 after transplant) [1]. Post hoc analyses demonstrated that participants were more likely to experience a higher frequency of supratherapeutic concentration (C) situations (defined as *C* > 20 ng/mL), particularly for CYP3A5 expressers in the genotype-based group (initial dose received 0.3 mg/kg/day) or CYP3A5 nonexpressers in the standard-dose group (initial dose received 0.2 mg/kg/day). The incidence of supratherapeutic Tac concentration in the two groups was 46.4% and 47.4%, respectively. This suggests that avoiding dose increases for CYP3A5 expressers could reduce supratherapeutic Tac exposure population-wide. In 2019 a French study [31] adjusted the CPIC guideline recommendations, lowering the starting dose of Tac for these genotypes in the prospective part of their study. Thus, the new regimen for the initial dose of Tac became the following: 0.10, 0.20 and 0.30 mg/kg/d for the genotypes CYP3A5*3/*3, CYP3A5*1/*3 and CYP3A5 *1/*1, respectively. The study showed that even when CYP3A5 non-expressers received the median initial dose of 0.1 mg/kg/day, nearly 40% of recipients still exhibited Tac overexposure. Our back-calculated doses (0.07–0.11, 0.14–0.21 and 0.20–0.29 mg/kg/d for the CYP3A5*3/*3, CYP3A5*1/*3 and CYP3A5 *1/*1 genotypes, respectively) compared to the French study (0.10, 0.20 and 0.30 mg/kg/d for the CYP3A5*3/*3, CYP3A5*1/*3 and CYP3A5 *1/*1 carriers, respectively) support the feasibility of CYP3A5-based dosing while suggesting that lower-than-conventional doses may be preferable.

One limitation of this study is that we did not assess the role of drug-drug interactions, particularly the inhibitors of CYP3A or P-glycoprotein which increase Tac concentration. Thus, our Tac concentration data may have been overestimated. Additionally, we did not collect data on Tac formulation use, nor did we stratify patients by CYP3A5 genotype to determine the distribution of formulations within each group. Differences in formulation may have affected dose requirements and Tac concentrations, representing a potential confounder. Future studies should quantify Tac formulation use and evaluate the impact of formulation switching on dosing to optimize pharmacogenetic strategies.

The strength of this study lies in its pharmacogenomic analysis of Asian KTx recipients, with a sufficient number of CYP3A5 *1/*1 carriers to yield robust conclusions. Our retrospective data revealed that the current dosing practices often result in high overexposure or toxic concentrations. Thus, we conclude that genotype-specific Tac doses are essential for pharmacogenetic optimization.

## Conclusions

In conclusion, we demonstrated that CYP3A5 variants significantly influence the required dose of Tac in KTx recipients, highlighting the need for an initial Tac dosing strategy based on CYP3A5 genotypes. Our research reveals the value of preemptive pharmacogenetic testing, with a back-calculated dosing formula in this Taiwanese cohort reducing Tac overexposure by 69%, paving the way for achieving adequate Tac therapeutic exposure in precision medicine. However, as this genotype-guided approach remains preliminary and specific to our population, future studies are required to incorporate additional Tac-associated genes and to validate this strategy prospectively in diverse cohorts.

## Supplementary Material

Supplementary File.docx

## Data Availability

All data generated or analyzed during this study are included in this published article.
