# Supplementary material for: Refining tacrolimus dosing through CYP3A5 pharmacogenetics in Taiwanese renal transplant recipients
Source: Ren Fail. 2025 Sep 11;47(1):2523567. doi: 10.1080/0886022X.2025.2523567 (PMC12427442; doi:10.1080/0886022X.2025.2523567)
Supplement: Supplementary File.docx [file IRNF_A_2523567_SM8698.docx]

| **Supplementary Table 1. Linear regression analysis to find independent predictors of stable Tac C_0_/D ratio on 3th month.** | | | |
| --- | --- | --- | --- |
| **Variable** | **Coefficient** | **standard error** | ***p* value** |
| Constant | 111.5 | 5.2 | <0.001* |
| CYP3A5*1/*1 | -69.6 | 11.5 | <0.001* |
| CYP3A5*1/*3 | -54.8 | 7.9 | <0.001* |
| R square=0.276; *p* value<0.001 *statistically significant. | | | |

| **Supplementary Table 2. Patient pharmacokinetic values during the study period according to CYP3A5 genotype.** | | | | | | | |
| --- | --- | --- | --- | --- | --- | --- | --- |
|  | CYP3A5*1/*1 (n=45) | | CYP3A5*1/*3 (n=162) | | CYP3A5*3/*3 (n=224) | | *p* value |
| **Day 0** |  |  |  |  |  |  |  |
| Tac dose (mg/daily) | 12.00 | (10-14) | 12.00 | (10-12) | 12.00 | (10-14) | 0.788 |
| Weight-adjusted TAC dose (mg/kg per day) | 0.19 | (0.15-0.2) | 0.18 | (0.15-0.2) | 0.19 | (0.16-0.2) | 0.900 |
| Tac level C_o_(ng/ml) | 7.70 | (5.95-10.15) | 8.60 | (6.5-10.4) | 9.75 | (7.7-12.55) | <0.001** |
| Dose-adjusted Tac level (ng/ml per mg/kg per day) | 43.05 | (30.91-55.72) | 47.55 | (36.01-64.12) | 59.53 | (44.09-76.73) | <0.001** |
| **Day 10** |  |  |  |  |  |  |  |
| Tac dose (mg/daily) | 15.00 | (10-19.5) | 10.00 | (10-14) | 8.00 | (6-9) | <0.001** |
| Weight-adjusted Tac dose (mg/kg per day) | 0.24 | (0.18-0.28) | 0.18 | (0.14-0.23) | 0.13 | (0.09-0.17) | <0.001** |
| Tac level C_o_(ng/ml) | 7.60 | (5.93-9.85) | 8.60 | (6.6-10.45) | 10.00 | (7.93-13.08) | <0.001** |
| Dose-adjusted Tac level (ng/ml per mg/kg per day) | 31.74 | (22.28-53.97) | 43.74 | (33.9-64.27) | 81.74 | (54.5-131.84) | <0.001** |
| **3 months** |  |  |  |  |  |  |  |
| Tac dose (mg/daily) | 10.00 | (10-12) | 8.00 | (6.5-10) | 5.00 | (3-6.5) | <0.001** |
| Weight-adjusted Tac dose (mg/kg per day) | 0.16 | (0.13-0.22) | 0.13 | (0.1-0.18) | 0.07 | (0.05-0.11) | <0.001** |
| Tac level C_o_(ng/ml) | 6.70 | (4.4-8.6) | 7.40 | (5.8-8.6) | 8.25 | (6.83-10.4) | <0.001** |
| Dose-adjusted Tac level (ng/ml per mg/kg per day) | 39.96 | (27.56-52.26) | 53.59 | (42.04-67.48) | 107.78 | (77.6-179.58) | <0.001** |
| **6 months** |  |  |  |  |  |  |  |
| Tac dose (mg/daily) | 10.00 | (8-12) | 8.00 | (6-10) | 4.00 | (3-6) | <0.001** |
| Weight-adjusted Tac dose (mg/kg per day) | 0.14 | (0.11-0.18) | 0.13 | (0.09-0.17) | 0.06 | (0.05-0.1) | <0.001** |
| Tac level C_o_(ng/ml) | 6.50 | (5-8.4) | 7.10 | (5.4-8.95) | 7.60 | (6.1-9.45) | 0.207 |
| Dose-adjusted Tac level (ng/ml per mg/kg per day) | 44.20 | (30.11-70.53) | 58.58 | (43.93-81.78) | 128.98 | (77.09-168.72) | <0.001** |
| **9 months** |  |  |  |  |  |  |  |
| Tac dose (mg/daily) | 9.25 | (6.75-10) | 7.00 | (5-10) | 4.00 | (2.75-5) | <0.001** |
| Weight-adjusted Tac dose (mg/kg per day) | 0.13 | (0.1-0.17) | 0.12 | (0.08-0.16) | 0.06 | (0.04-0.09) | <0.001** |
| Tac level C_o_(ng/ml) | 6.35 | (4.53-7.83) | 7.00 | (4.8-8) | 7.00 | (5.55-9.15) | 0.058 |
| Dose-adjusted Tac level (ng/ml per mg/kg per day) | 48.64 | (36.14-63.58) | 54.72 | (42.17-82.81) | 122.47 | (91.63-180.13) | <0.001** |
| **1 year** |  |  |  |  |  |  |  |
| Tac dose (mg/daily) | 9.00 | (7-10) | 6.00 | (5-8) | 4.00 | (2.5-5) | <0.001** |
| Weight-adjusted Tac dose (mg/kg per day) | 6.90 | (5.95-7.75) | 6.20 | (4.8-8.1) | 7.40 | (5.3-8.7) | 0.026* |
| Tac level C_o_(ng/ml) | 0.13 | (0.11-0.18) | 0.12 | (0.07-0.15) | 0.06 | (0.04-0.08) | <0.001** |
| Dose-adjusted Tac level (ng/ml per mg/kg per day) | 47.20 | (33.55-68.01) | 57.40 | (42.13-74.9) | 121.18 | (80.74-186.13) | <0.001** |
| Chi-Square test. Kruskal Wallis test. **p*<0.05, ***p*<0.01. Non-normal variables were reported as median (IQR). | | | | | | | |
